# Effect of sexual health education on sexual function and satisfaction of menopausal migrant women: an application of the theory of planned behavior

**DOI:** 10.1186/s12889-024-19162-w

**Published:** 2024-06-18

**Authors:** Maryam Mohammadi, Nooshin Peyman, Mohadese Hossainabadi, Vahid Ghavami, Hadi Tehrani

**Affiliations:** 1 Social Determinants of Health Research Center, Mashhad University of Medical Sciences, Mashhad, Iran; 2 MSC student of Health Education and Health Promotion, Student Research Committee, Mashhad University of Medical Sciences, Mashhad, Iran; 3 Department of Biostatistics, School of Health, Mashhad University of Medical Sciences, Mashhad, Iran; 4 Department of Health Education and Health Promotion, School of Health, Mashhad University of Medical Sciences, Mashhad, Iran

**Keywords:** Theory of planned behavior, Sexual function, Marital satisfaction, Postmenopausal women, Migrant women

## Abstract

**Objectives:**

This study investigated the effect of an intervention based on the theory of planned behavior on sexual function and satisfaction of migrant women during menopause in Iran.

**Methods:**

This quasi-experimental study was conducted on 88 migrant and menopausal women in Iran. Sampling was performed using the multistage methods in four health care centers. The educational program based on the theory of planned behavior was held in 4 sessions of 90 min for 4 weeks in the intervention group. The final evaluation of the intervention was performed immediately, and the follow-up stage (3 months after the intervention) by completing questionnaires in two groups. The data was analyzed with SPSS 20 software with statistical tests of mean and standard deviation, Manwitney, Frideman, Generalized Estimating Equations.

**Results:**

The results showed that after the intervention, the mean score of sexual function in the intervention group increased from 16.53 ± 2.68 before to 17.52 ± 2.90 immediately and 17.38 ± 2.81 in follow up stage (*p* < 0.05). But in the control group, this score was not statistically significant during the study stages (*p* > 0.05).

**Conclusion:**

The results indicate that the intervention based on the theory of planned behavior is effective in sexual function and satisfaction with the married life of migrant women during menopause. but to change the sexual function, studies with a longer duration and also the use of other educational models are suggested.

## Introduction

Although sexual complaints are common throughout a person’s sexual life, menopausal women are more susceptible to these disorders, and the likelihood of experiencing sexual dysfunction is higher in menopausal women [1, 2]. Costello et al. reported a 50% prevalence of sexual dysfunction in postmenopausal women, and the prevalence of these problems increases with age [3]. In a survey conducted in six European countries, one-third (34%) of respondents reported experiencing a decrease in libido during menopause, while 53% stated that their sexual interest had decreased [4, 5]. According to Nicolosi et al., 39% of women are affected by at least one sexual dysfunction [6]. The prevalence of sexual disorders during or after menopause in Iran is also reported to be approximately 72.4–90% [7, 8]. The statistics stated in various studies prove the importance of paying attention to sexual health because sexual instinct is one of an innate human need [9].

According to a report by the World Health Organization, sex education programs are considered a necessity both for people who have not yet started their sexual activity and for people who have started their sexual activity [10]. Considering the ever-increasing growth of the elderly population, it is necessary to pay attention to marital satisfaction in this age group [11]. Research on women who migrate from their country of origin and their experiences of menopause and life after menopause is scarce; however, growing evidence shows that migration affects all aspects of women’s health and well-being. During menopause, migrant women are vulnerable because they simultaneously experience stressful factors related to their connection to the new society [12]. Migration and menopause are areas that potentially increase women’s vulnerability [13]. The Khorasan-Razavi province and Mashhad city are also important centers for accepting foreign migrants because of their proximity to the country’s eastern borders. Khorasan-Razavi province has a common border with Turkmenistan for about 531 km from the east and northeast, and 3 cities located in the east of the province share a common border with Afghanistan for 302 km [14].

The results of most studies are based on the principle that training based on theories can have a positive effect on increasing the capabilities of menopausal women and improving their sexual function. Based on these studies, it has been shown that the planned behavior model is suitable for education in the field of menopause [15, 16].

The theory of planned behavior was developed by Ajzen and Fishbein in 1980. This theory is a social-cognitive model of value expectation that considers intention as the main determinant of behavior. This theory states that attitude (the degree to which a person has a positive or negative evaluation of performing a behavior), subjective norms (normative influences; perceived social pressures to perform or not perform a behavior), and perceived behavioral control (perceived difficulty) or the ease of performing a certain behavior) have an effect on the intention and directly affect the behavior. This theory pays attention to social factors such as social norms and the motivation to obey important people, and for this reason, many studies consider it an important factor in accepting desirable sexual functions [17–19].

Due to the fact that immigrants in the host country have relatively less welfare facilities and social services, they are more exposed to harm, which leads to disturbances in psychological, socio-cultural adjustment and can affect various aspects of immigrants’ health. including their sexual health [20–22]. Also, since little research has been done to identify factors affecting the quality of sexual life and the difference in the vulnerability of different immigrant populations. Therefore, the present study was conducted to determine the effect of educational intervention based on the theory of planned behavior on sexual function and marital satisfaction of migrant women during menopause.

## Methods

### Study design and sampling

**Fig. 1:**
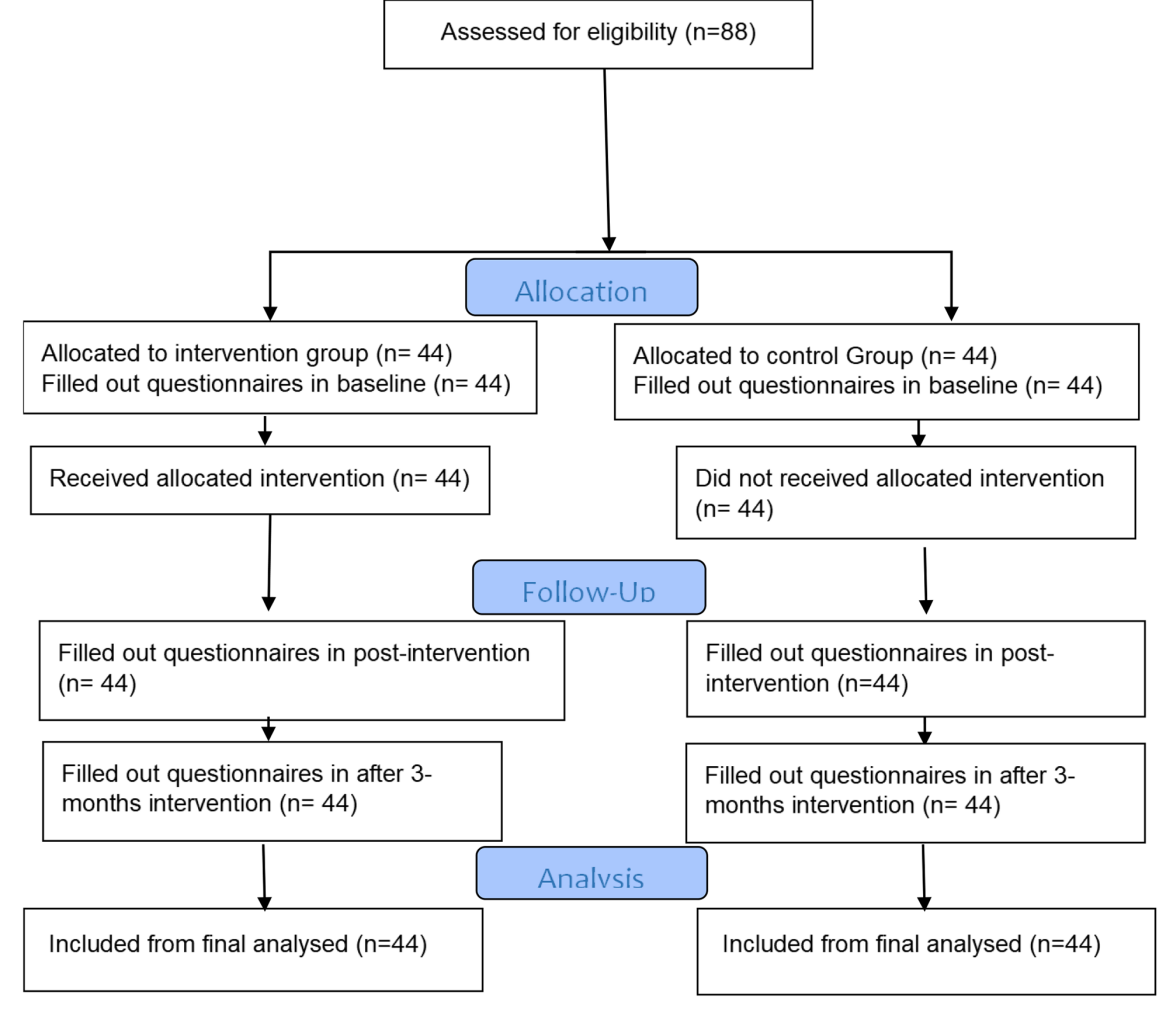
Study flow chart

A quasi-experimental study was conducted on 46-65-year-old married migrant women (foreign nationals) who were referred to a health center in Mashhad city of Iran. The research population included 235 immigrant women. According to previous studies [23] and considering a power of 80, a confidence interval of 95%, and an effect size of 0.63, the sample size was estimated to be 40 people for each group. Taking into account a 10% sample loss, the final sample size for each group was estimated to be 44 people. However, in the study, 88 migrant women during menopause based on inclusion criteria (Immigrant women, consent to participate in the study, age 46–65 years, married and living Being stable with a spouse, at least 1 and at most 5 years past menopause, Literacy for reading and writing, Proficiency in Persian language were included and randomly divided into two interventions (*n* = 44) and control group (*n* = 44) (Fig. 1).

Sampling was done in a multi-stage method, first, one center was selected from among 5 health centers in Mashhad city by cluster random method, and then 4 Comprehensive database of health services were selected by simple random lottery method, and the necessary samples were selected by simple random from any comprehensive health service base according to Flowchart 1.

### Educational intervention

The educational intervention was carried out in 4 sessions of 90 min as follows (Table 1). It should be noted that in this study, we did not have a program for the control group, but at the end of the intervention program, an educational package was provided to them, and for those who were willing, the educational program was implemented.

**Table 1:** Educational intervention based on Bloom’s cognitive-emotional and psychomotor classification

Session	Title	content	equipment	Intervention Method	Time
1	Menopause and related symptoms	• Definition of menopause • Complications caused by menopause • Symptoms of menopause and its treatment methods • Methods that reduce complications caused by menopause in women. • Preparation of material about one of the common symptoms of menopause and its treatment method	Computer, data projector Whiteboard and marker Booklets	Lecture, question and answer brainstorming	90 min
2	Male and female reproductive system	• Female internal and external genitals, male genitalia • Sexually sensitive areas of women and men • Stages of the normal sexual response cycle • Changes in women’s sexual cycle due to menopause • Sexual dysfunctions	Computer, data projector Whiteboard and marker	Lecture, question and answer	90 min
3	Overcoming disorders of sexual function of menopausal women	• Overcoming disorders of sexual function of menopausal women • Positive points during menopause • Ways to deal with low libido	Computer, data projector Whiteboard and marker, Pamphlet	Lecture, question and answer brainstorming group discussion	90 min
4	Communication skills with spouse	• Communication skills with spouse • Maintain enthusiasm and interest • Barriers to not expressing emotions • Ways to resolve conflicts • How to criticize and accept criticism • Recommendations to increase marital satisfaction	Computer, data projector Whiteboard and marker	Lecture, question and answer group discussion.	90 min

### Data collection tools

The data collected tool was the following questionnaire, which was completed via Self-reported by the research samples before, immediately and follow up stage.

#### Demographic information

includes 9 questions about women’s age, Spouse’s age, marriage age, duration of menopause, gravida, number of children, number of unmarried children, number of sex per month.

### The questionnaire made by the researchers based on the model structures

**Attitude questions** include 16 questions that were measured on a five-point Likert scale. The minimum and maximum marks for the questions in this section were 1 and 5, respectively. In total, the points obtained in this section were calculated between 16 and 80. **Subjective Norms questions** included 7 questions that were measured on a five-point Likert scale. The minimum and maximum marks for the questions in this section were 1 and 5, respectively. In total, the points obtained in this section were calculated between 7 and 35. **Perceived behavioral control questions** included 4 questions that were measured on a five-point Likert scale. The minimum and maximum marks for the questions in this section were 1 and 5, respectively. In total, the points obtained in this section were calculated between 4 and 20. **Behavioral intention questions** included 4 questions that were measured on a five-point Likert scale. The minimum and maximum marks for the questions in this section were 1 and 5, respectively. In total, the points obtained in this section were calculated between 4 and 20. And **Behavioral questions**, including 3 questions with two options (answer yes score 1 and answer no score zero). The minimum score was 0 and the maximum score was 3.

#### Sexual function questionnaire

this questionnaire measures women’s sexual function in 6 independent areas of sexual desire, sexual excitement, sexual moisture, orgasm, satisfaction and painful intercourse. In total, the points obtained in this section were calculated between 2 and 36. Validity and reliability of this questionnaire in Iran has been confirmed in Mohammadi’s study [24].

#### Marital satisfaction questionnaire

It has 47 questions in the field of marital satisfaction, personality issues, relationship with spouse, conflict resolution, financial management, leisure time, sex, children and parenting, family and friends, and orientation. It was scored on a 5-point Likert scale from one to five. According to the selected options, the maximum score for the subject is 235. A score higher than 60 indicates complete marital satisfaction, scores 40–60 indicate partial marital satisfaction, and a score lower than 40 indicates dissatisfaction with the marital relationship [25, 26]. The validity and reliability of these tools were confirmed in the previous studies [27–30]. The validity and reliability of this questionnaire has been confirmed as a valid in the previous researches conducted in this field [25, 26].

The validity and reliability of the questionnaire was confirmed by determining content validity index and content validity ratio (by 10 professors of Mashhad University of Medical Sciences) (CVI = 0.78, CVR = 0.85) and Cronbach’s alpha was obtained between 0.75 and 0.98 for different parts and 0.95 for the whole instrument.

The participants in the control and intervention groups completed the questionnaires through interviews at three time points: before the intervention, immediately after the intervention, and during the 3-month follow-up.

### Data analysis

Completed questionnaires were entered into SPSS 20 software. For this purpose, initially, for data analysis, the normality of quantitative variables was determined using the Kolmogorov-Smirnov test. The Independent Student’s t-test, Mann-Whitney and Chi-square statistical test were used to test homogeneity. Descriptive statistics were used to describe the characteristics of the research unit. In all tests, a significant level of 0.05 was considered.

## Results

In this study, 88 migrant women during menopause with a mean age of 55.95 ± 5.93 were included in the study. In examining the demographic characteristics of the research samples, there was no significant difference between the research samples in terms of demographic variables(*p* > 0.05), except for the time past of menopause and the amount of sexual intercourse per month(*p* < 0.05). (Table 2).

**Table 2:** Comparison of the demographic characteristics of the studied in control and intervention groups

Variable	Control group	Intervention group	Test result*
	SD ± M	SD ± M	
women’s age	51.78 ± 3.52	53.1 ± 4.97	Z = 1.083 *P* = 0.279
Marriage age	14.71 ± 2.18	14.67 ± 2.43	Z = 0.263 *P* = 0.792
duration of menopause	3.32 ± 2.73	4.97 ± 3.78	Z = 1.948 *P* = 0.051
Gravida	5.51 ± 1.64	5.43 ± 1.47	Z = 0.186 *P* = 0.853
Number of children	5.05 ± 1.61	4.83 ± 1.56	Z = 0.649 *P* = 0.517
Number of unmarried children	2.17 ± 1.28	2.4 ± 1.15	Z = 0.708 *P* = 0.479
Number of sexes per month	2.66 ± 1.73	3.38 ± 1.85	Z = 2.029 *P* = 0.043
Spouse’s age	55 ± 4.2	56.43 ± 5.1	Z = 1.267 *P* = 0.205

* Manwitney

The mean comparison of the research variables based on the planned behavior model before, immediate and follow-up stages in the control and intervention groups is shown in Table 3. As can be seen, the mean sexual function in the intervention group increased from 16.43 ± 2.68 before the intervention to 17.52 ± 2.90 immediate and 17.38 ± 2.81 in follow up stage (*p* < 0.05), but this increase was not observed in the control group(*p* > 0.05).

The mean of marital satisfaction in the intervention group increased from 129.9 ± 11.19 before the intervention to 132.05 ± 11.71 immediately after the intervention and 133.14 ± 11.33 in follow up stage. This increase was not observed in the control group(*p* > 0.05). (Table 3)

The results of the present study indicate that there was a significant difference in the attitude score in the intervention group before 53.1 ± 5.32, immediate 54.33 ± 5.1 and follow up stage 54.07 ± 5.17, and this score was not significant in the control group (*p* > 0.05). (Table 3)

The results obtained from comparing the mean of subjective norms in the intervention group showed that the score of subjective norms before the intervention, 22.57 ± 3.08, had a significant difference immediately after the intervention, 23.21 ± 2.93 and follow up stage 23.02 ± 2.81. (*p* < 0.05) (Table 3).

The results obtained from comparing the mean of perceived behavior control in the intervention group also showed that before the intervention 13.19 ± 2.17 had a significant difference (*p* < 0.05) with that immediately after the intervention 13.67 ± 2.07, but it did not show a significant difference with follow up stage 13.40 ± 2.1. (*p* > 0.05)

The results of the study showed there was a significant difference between the sexual function score before the intervention 16.43 ± 2.68, immediate 17.52 ± 2.90 and follow up stage 17.38 ± 2.81 in the intervention group. This increase was not observed in the control group(*p* > 0.05). (Table 3)

**Fig. 2:**
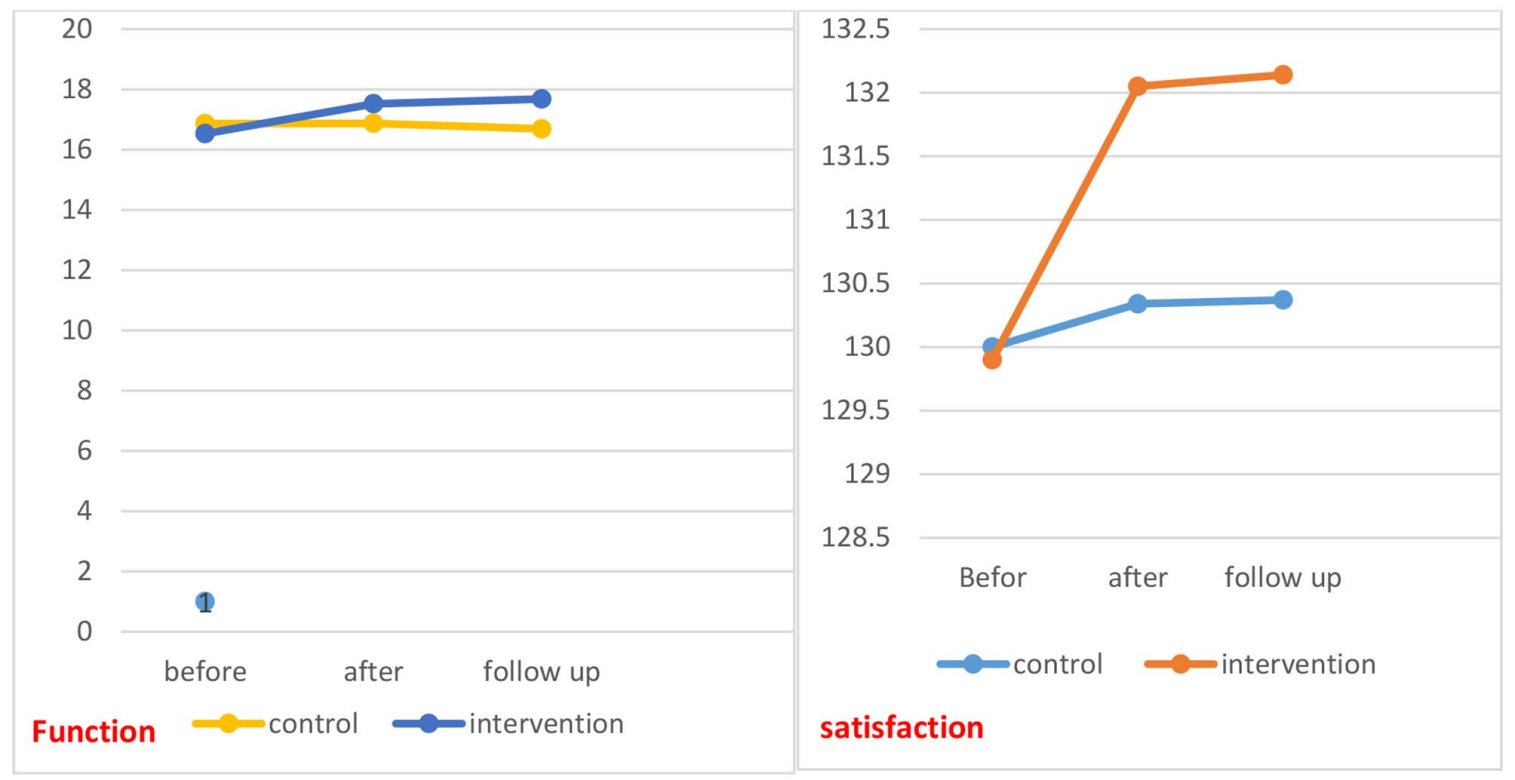
Satisfaction and Function with married life before, immediately and follow up stages in the control and intervention groups

Also, the results of this study regarding marital satisfaction showed that it was increased during the intervention stages, so that the satisfaction score in the stage before the educational intervention was (129.9 ± 11.19) increased to (132.05 ± 11.71) immediately and (133.14 ± 11.33) follow up stage and this difference was statistically significant. *p* < 0.05) but no significant difference was observed in the control group (Table 3; Fig. 2).

**Table 3:** Comparison of mean research variables based on the model of planned behavior before, immediately and follow up in the control and intervention groups

Time\Variables & groups		Intervention			In-group **test	Difference mean between the immediate and before the intervention	Ddifference mean between the follow up and before the intervention
		before	immediate	Follow up			
		SD ± M	SD ± M	SD ± M			
Attitude towards sex	Intervention	53.1 ± 5.32	54.33 ± 5.1	54.07 ± 5.17	*P* < 0.001 F = 43.43	1.23	0.97
	control	52.95 ± 6.75	52.76 ± 6.78	52.29 ± 6.43	*P* < 0.001 F = 18.27	-0.29	-0.65
Intergroup test*		*P* = 0.531 Z = 0.626	*P* = 0.234 t = 1.199	*P* = 0.076 Z = 1.772			
Subjective Norms	intervention	22.57 ± 3.08	23.21 ± 2.93	23.02 ± 2.81	*P* < 0.001 F = 23.17	0.64	0.45
	control	21.37 ± 3.21	21.41 ± 3.1	20.9 ± 3.09	*P* < 0.001 F = 19.39	0.45	-0.46
Intergroup test*		*P* = 0.042 Z = 2.036	*P* = 0.006 Z = 2.757	*P* = 0.001 Z = 3.219			
Perceived control behavior	intervention	13.19 ± 2.17	13.67 ± 2.07	13.40 ± 2.1	*P* < 0.001 F = 18.60	0.47	0.21
	control	13.51 ± 2.87	13.44 ± 2.93	13.29 ± 2.97	*P* = 0.015 F = 8.40	0.68	-0.22
Intergroup test*		*P* = 0.948 Z = 0.065	*P* = 0.170 Z = 1.373	*P* = 0.195 Z = 1.297			
Intention to have sex	intervention	13.45 ± 1.73	14.26 ± 1.61	13.93 ± 1.72	*P* < 0.001 F = 40.26	0.81	0.47
	control	13.29 ± 1.5	13.27 ± 1.43	13.12 ± 1.27	*P* = 0.030 F = 7	0.23	-0.17
Intergroup test*		*P* = 0.856 Z = 0.182	*P* = 0.006 Z = 2.745	*P* = 0.026 Z = 2.229			
sexual function	intervention	16.43 ± 2.68	17.52 ± 2.90	17.38 ± 2.81	*P* < 0.001 F = 61.52	1.1	0.95
	control	16.86 ± 2.67	16.87 ± 2.69	16.69 ± 2.65	*P* = 0.029 F = 7.10	0.01	-0.17
Intergroup test*		*P* = 0.464 T = 0.735	*P* = 0.245 Z = 0.245	*P* = 0.180 Z = 1.339			
Marital satisfaction	intervention	129.9 ± 11.19	132.05 ± 11.71	133.14 ± 11.33	*P* < 0.001 F = 56.35	2.15	3.24
	control	130.22 ± 10.81	130.34 ± 10.75	130.37 ± 10.57	*P* = 0.752 F = 0.56	0.12	0.15
Intergroup test*		*P* = 0.844 Z = 0.196	*P* = 0.514 Z = 0.652	*P* = 0.430 Z = 0.789			

* Manwitney, **Frideman

The results of this study in relation to the dimensions of sexual function showed that this score increased in the stages of the study (before 16.43 ± 2.68, immediately 17.52 ± 2.90 and follow-up17.38 ± 2.81) in the intervention group, but this increase was not observed in the control group. (Table 4; Fig. 2). The dimensions of sexual performance during the study in the control and intervention groups are listed separately in Table 4.

**Table 4:** Mean sexual function and its dimensions before, immediately and follow up the intervention in the intervention and control groups

Time\Variables & groups		Intervention			In group test**
		before	immediately	Follow up	
		SD ± M	SD ± M	SD ± M	
sexual function	Intervention	16.43 ± 2.68	17.52 ± 2.90	17.38 ± 2.81	*P* < 0.001 F = 61.52
	control	16.86 ± 2.67	16.87 ± 2.69	16.69 ± 2.65	*P* = 0.029 F = 7.10
Intergroup test*		*P* = 0.464 t = 0.735	*P* = 0.245 Z = 1.162	*P* = 0.180 Z = 1.339	
sexual excitement	intervention	1.88 ± 0.46	1.97 ± 0.47	1.94 ± 0.46	*P* = 0.001 F = 13.35
	control	1.88 ± 0.49	1.88 ± 0.48	1.84 ± 0.48	*P* = 0.038 F = 6.54
Intergroup test*		*P* = 0.952 Z = 0.0660	*P* = 0.347 Z = 0.940	*P* = 0.262 Z = 1.122	
sexual moisture	intervention	2.5 ± 0.63	2.55 ± 0.61	2.54 ± 0.61	*P* < 0.001 F = 15.21
	control	2.45 ± 0.51	2.48 ± 0.48	2.39 ± 0.48	*P* = 0.014 F = 8.6
Intergroup test*		*P* = 0.819 Z = 0.229	*P* = 0.661 Z = 0.438	*P* = 0.168 Z = 1.380	
orgasm	intervention	2.11 ± 0.65	2.25 ± 0.70	2.22 ± 0.67	*P* < 0.001 F = 15.85
	control	2.18 ± 0.63	2.17 ± 0.64	2.13 ± 0.62	*P* = 0.0140 F = 3.93
Intergroup test*		*P* = 0.454 Z = 0.650	*P* = 0.520 Z = 0.644	*P* = 0.478 Z = 0.710	
sexual desire	intervention	4.33 ± 0.77	4.64 ± 0.61	4.60 ± 0.61	*P* < 0.001 F = 33.31
	control	4.46 ± 0.52	4.47 ± 0.53	4.47 ± 0.50	*P* = 0.939 F = 0.125
Intergroup test*		*P* = 0.447 T = 0.760	*P* = 0.124 Z = 1.540	*P* = 0.198 Z = 1.288	
painful intercourse	intervention	3.74 ± 1.13	4.1 ± 0.88	4.06 ± 0.92	*P* < 0.001 F = 39.41
	control	4.15 ± 1.12	4.1 ± 1.16	4.09 ± 1.13	*P* = 0.018 F = 8
Intergroup test*		*P* = 0.100 Z = 1.645	*P* = 0.864 Z = 0.171	*P* = 0.945 Z = 0.069	

* Manwitney, **Frideman

The GEE (Generalized Estimating Equation) model was used to investigate the effect of intervention on sexual intercourse per month and past time of the menopause (because of the significant difference in the two study groups).

In this model, changes in sexual function compared to before the intervention were entered into the GEE model as response variables. Based on the results obtained from fitting the model, the mean score of changes in sexual function (compared to before the intervention) in the intervention group was 1.09 more than the control group. Also, these changes immediately after the intervention were significantly more than the follow-up stage (*P* = 0.003). The effect of the amount of sexual intercourse per month also had a significant effect on the model, so that by increasing the amount of sexual intercourse per month once, the amount of changes in sexual function increased by 0.157. According to the above model, the effect of none of the constructs of the theory of planned behavior was significant (Table 5).

Next, to investigate the effect of education on marital satisfaction while controlling the constructs of the Theory of Planned Behavior model and the variables of time of the menopause and the amount of sexual intercourse per month (due to the significant difference in the two study groups) and also the effect of time from the GEE (Generalized Estimating Equation) model used. In this model, changes in marital satisfaction compared to before the intervention were entered into the GEE model as response variables. Based on the results obtained from fitting the super-mean model, the score of changes in marital satisfaction (compared to before the intervention) in the intervention group was 2.41 more than the control group. Based on this model, the effect of study time (*P* = 0.629), the number of sexes per month (*P* = 0.379) and none of the constructs of the theory of planned behavior were significant (Table 5).

**Table 5:** The results of fitting the GEE model to simultaneously examine the relationship between variables on changes in sexual performance and marital satisfaction

Variable		sexual function			marital satisfaction		
		Regression coefficient	Standard error of measurement	*P*-value	Regression coefficient	Standard error of measurement	*P*-value
group	Intervention	1.099	0.201	< 0.001	2.412	0.458	< 0.001
	control	-	-	-	-	-	-
Time	immediately	0.165	0.056	0.003	0.078	0.162	0.629
	Follow up	-	-	-	-	-	-
Number of sex per month		0.157	0.044	< 0.001	0.379	0.11	-0.097
Age of menopause		-0.049	0.026	0.068	0.249	0.083	0.096
attitude		-0.041	0.037	0.270	0.537	0.116	-0.072
Subjective Norms		0.212	0.052	0.065	0.825	0.175	0.039
Perceived behavioral control		0.075	0.061	-0.11	0.191	0.236	-0.31
behavioral intention		0.637	0.077	0.037	0.165	0.187	0.26

* Generalized Estimating Equations

## Discussion

The results of the present study showed that the educational intervention increased the sexual function in the intervention group during the study. In line with the results of the current research, Ebrahim et al. (28) showed in their study that education has a positive effect on awareness, intention, and sexual function.

Regarding marital satisfaction in the control group during the study, no difference was observed, but in the intervention group, the mean satisfaction was increased the marital life in the intervention group went from 129.9 ± 11.19 before the intervention to 132.05 ± 11.71 immediately after the intervention and the 133.14 ± 11.33 in follow up stage. Therefore, educational intervention has increased the score of satisfaction with married life in the intervention group. A study conducted by Shahsiah et al. [31] under the title “Effect of sex education on improving marital satisfaction of couples in Isfahan city” showed that the educational intervention caused the satisfaction score of married life to increase. This study is consistent with the findings of the current research.

Also, in Iran, Mohammadi et al. found inconsistent results compared to this research. One of the reasons for this is the unique challenges faced by migrant women during menopause. Research on women who migrate from their country of origin and their experiences of menopause and life after menopause is limited, yet there is increasing evidence that migration impacts all aspects of women’s health and well-being. During menopause, migrant women are vulnerable because they simultaneously experience stressful factors related to their connection to the new society [21]. Migration and menopause are areas that potentially increase women’s vulnerability [22].

The results of the present study indicate that there was a significant difference between the attitude score before the intervention and immediately after the intervention and the follow-up stage in the intervention group, and this score was not significant in the control group. Therefore, the educational intervention designed in this research has increased the attitude of the training group. Rifai Shirpak et al. [32] In a study entitled “Sexual education in women referring to Tehran health centers”, there examined the attitude in their sex education program was a significant difference in attitude of intervention and control group. The results of this study are consistent with the findings of the present study. Tabatabai et al. [30] in research titled “Effect of educational intervention based on the theory of planned behavior on the physical activity of employees of the health center of Kerman province”, stated that the mean attitude variable score of the training group after the intervention did not have a statistically significant difference with the control group. The present study was not consistent with this study. According to the researcher, the possible reasons for this include less involvement of the studied group in learning, fewer self-made discussions and the use of passive educational methods in the study of Tabatabaei [30].

The results of the present study showed that the educational intervention increased the score of Subjective Norms in the intervention group. Deborah Konyak and colleagues [33] In research titled “Predictors of high-risk functions among teenage mothers in an immune system deficiency prevention program”, stated that after 3 and 6 months of follow-up, there was a statistically significant difference in the Subjective Norms of the intervention group after the intervention. The findings of this study are consistent with the results of the present study.

The results obtained from the comparison of the perceived behavior control score in the intervention group showed that there was a significant difference between the perceived behavior control score before the intervention and immediately after the intervention, but no significant difference was observed in the follow-up phase. This means that the educational intervention increased the perceived behavior control score in the intervention group. Understanding the control of behavior and feeling under the will and agency of action for behavior is one of the important factors of establishing sexual function and having higher marital satisfaction. Hatifnia et al. [34] showed a significant difference between the perceived behavior control score before and immediately after the intervention.

In the present study, educational intervention increased behavioral intention in the intervention group. Mausbach T. et al. [35] also showed in a study titled “Predictors of safe sexual function and protected sex among HIV-negative methamphetamine users using the theory of planned behavior” that among users’ Heterosexual methamphetamine, the theory of planned behavior has a good theory for predicting the intention of safe sexual function and the constructs of this theory have been effective in reducing the use of methamphetamine. Tabatabai et al. [30] In research entitled “Effect of an educational intervention based on the theory of planned behavior on the physical activity of Kerman health center employees”, stated that after the intervention, in the mean scores, awareness and behavioral intention of the intervention group increased significantly.

### Limitations

The questionnaire was self-reported and the researcher assumed that the participants would answer honestly. The present study is related to migrant women in urban health centers who are in menopause and meet the entry and exit criteria defined in this study, and its generalizability is limited to the statistical population defined in this study.

The effectiveness of participants in the research from other sources other than the training course.

## Conclusion

The results of this research showed that the educational intervention based on the theory of planned behavior, considering the important factors affecting sexual function through model variables, improves sexual function and marital satisfaction of migrant women during menopause but to change the sexual function, studies with a longer duration and also the use of other educational models are suggested.

## Data Availability

Data will be provided by the corresponding author upon request.
